# Body mass index is a barrier to obesity treatment

**DOI:** 10.3389/fendo.2024.1444568

**Published:** 2024-08-01

**Authors:** Geoffrey C. Chin, Adam W. Potter, Karl E. Friedl

**Affiliations:** ^1^ Thermal and Mountain Medicine Division, United States (U.S.) Army Research Institute of Environmental Medicine, Natick, MA, United States; ^2^ Office of the Senior Scientist, United States (U.S.) Army Research Institute of Environmental Medicine, Natick, MA, United States

**Keywords:** body fat, body mass index, BMI, metabolic syndrome, obesity

## Abstract

The Food and Drug Administration’s (FDA) obesity drug guidance is set on the basis of body mass index (BMI), with thresholds of either BMI ≥30 or BMI ≥27 kg/m^2^ with weight-related comorbidities. While BMI is associated with obesity-related health outcomes, there are known limitations to use as a direct measure of body fat or metabolic health, and the American Medical Association has highlighted limitations of BMI in assessing individual obesity risks. BMI thresholds impose a barrier to treatment. In a sample from the NHANES dataset (n=6,646 men and women), 36% of individuals with metabolic syndrome (MetS) may not be eligible for obesity pharmacotherapy. This analysis provides quantifiable justification for refinement of the BMI treatment criteria with a more holistic assessment of individual obesity-related disease risk.

## Introduction

It has been nearly 25 years since the Centers for Disease Control and Prevention (CDC) declared obesity an epidemic (1). Recent approvals of drugs for treatment of obesity are leading to a new age of therapeutic options. In 2015, the Food and Drug Administration (FDA) approved its first set of pharmacotherapy options for the treatment of obesity (liraglutide, phentermine/topiramate, and naltrexone/bupropion). This milestone marked a shift into an era of actual clinical treatment options for obesity. Indeed, liraglutide combined with exercise and low-calorie diet was very effective in treating metabolic syndrome (2, 3).

Existing FDA guidance for obesity treatment requires use of body mass index (BMI; kg/m^2^) for eligibility. Current FDA obesity drug guidance targets persons with BMI ≥30 or those BMI ≥27 with at least one accompanying weight-related comorbidity (e.g., hypertension, type 2 diabetes mellitus (T2DM), or dyslipidemia) (4). However, ten years since recognizing obesity as a disease, the American Medical Association (AMA) has now highlighted the limitations in BMI for assessing individual obesity risks, discouraging reliance on BMI to deny appropriate insurance reimbursement (5). BMI is a limited surrogate indicator of excess body fat and, at an individual level, BMI is a relatively poor predictor of adiposity or risk of metabolic disease (6).

Metabolic syndrome (MetS) is a primary obesity-related concern, with a doubling major cardiovascular disease outcomes and an increase all-cause mortality 1.5-fold, and with even higher risks among women (7). Since weight loss of 5-10% is shown to significantly improve abnormal components of MetS, the FDA uses 5% weight loss as the benchmark for obesity medications (4). However, in a systematic review of 950,000 MetS individuals, mean BMI ranged from 22-33 kg/m^2^ (7). Another landmark analysis of 195 countries estimated that 40% of cardiovascular deaths and 39% of high BMI-related deaths occurred among persons with BMI <30 (8). Rigid interpretation of package inserts may exclude many from obesity pharmacotherapy already at high risk for morbidity and mortality.

This analysis provides quantitative data and interpretations for improving these existing guidelines to ensure maximal benefit is allowed for those requiring treatment.

## Materials and methods

### Study design and sample population

A correlational analysis of data from cross sectional sampling of the US population via the National Health and Nutrition Examination Survey (NHANES) public use datasets was used for this study (9). The NHANES provides a demographically representative sampling of the US population that is collected continuously and collated into two-year datasets. The NHANES has been approved by the NCHS Research Ethics Review Board, therefore this analysis did not require a separate regulatory approval. Each participant within the NHANES study provided written informed consent prior to assessments (9).

As the FDA approved the first set of pharmacotherapy options for treatment of obesity in 2015, NHANES data was obtained from adults surveyed during 2015-2020, to evaluate for MetS prevalence and FDA obesity medication eligibility. An analysis was conducted using data from 6,646 adults (3,219 men, 3,427 women), evaluated for metabolic syndrome (MetS) prevalence and FDA obesity medication eligibility.

Demographic data was stratified by age, sex, race, and ethnicity. Using revised National Cholesterol Education Program Adult Treatment Panel III criteria (10), where MetS was defined as presence of at least 3 components: waist circumference (WC; cm) men ≥102 or women ≥88, blood pressure (BP; mmHg) systolic ≥130 or diastolic ≥85 or hypertension treatment, triglycerides (TG; mg/dL) ≥150 or dyslipidemia treatment, high-density lipoprotein cholesterol (HDL-C; mg/dL) <40 in men or <50 in women, and glucose (mg/dL) ≥100 or dysglycemia treatment. Participants with BMI ≥30 or ≥27 with hypertension, T2DM, or dyslipidemia were FDA-eligible for obesity medications (4). The consort flow diagram is presented in **Supplementary Figure S1**.

### Statistical analyses

Statistical analyses were conducted using a combination of SAS version 9.4 (SAS Institute), SPSS version 28.01.1 (IBM Corporation), and Excel (Microsoft Corporation). Descriptive statistics are shown as mean ± standard deviation, or by number of incidences. Calculations were made for false negative (FN), false positive (FP), true positive (TP), and true negative (TN) observations. McNemar’s test was used to evaluate discordancy between criteria with statistical significance set at *p <*0.05 (11). Cohen’s kappa test was used to evaluate consistency between criteria with *κ* <0.40, between 0.40-0.75, and >0.75 denoting marginal, good, and excellent reproducibility, respectively (12). Each of these methods were chosen to describe the 2x2 data as both a contingency table (McNemar’s) and confusion matrix (Cohen’s).

## Results

Data analyses were conducted on 6,646 adults (3,219 men, 3,427 women), including self-reported race/ethnicity as 34% non-Hispanic white, 26% Hispanic, 24% non-Hispanic black, 12% non-Hispanic Asian, and 5% non-Hispanic multiple. Descriptive statistics are shown in **Supplementary Table S1**.

**Figure 1:**
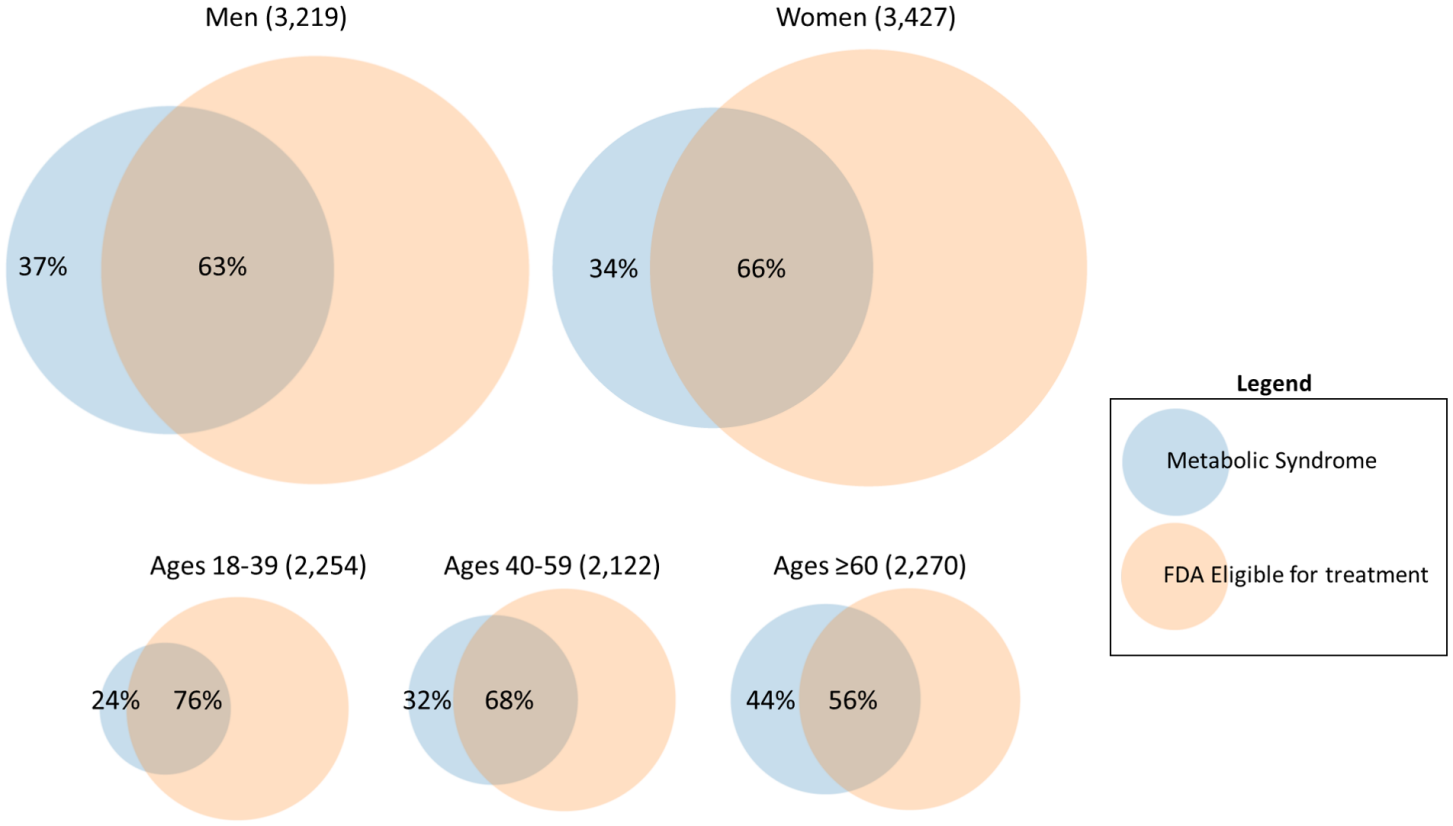
Overall agreement between FDA obesity medication eligibility criteria for individuals with metabolic syndrome, by sex and age groups.

**Table 1** describes the main analyses and **Figure 1** shows the overall agreement by sex and age groups. Of the 6,646 sample, 37% and 41% met MetS and FDA criteria, respectively. However, of those that met the MetS criteria, 37% of men and 34% of women did not meet the FDA criteria for pharmacological treatment of obesity. Analyses of the total population showed marginal overall agreement (*κ* =0.37) and significant discordance (*p <*0.001), and approximately 36% of the total MetS individuals did not meet FDA eligibility. Analyses of by age groups (18-39, 40-59, and ≥60), and for women showed marginal agreement (0.31-0.40) and significant discordance (*p <*0.001). However, for men analyses showed marginal overall agreement (*κ* =0.33) and non-significant discordance (*p* =0.476); while results were mixed for race/ethnicity subgroups (non-Hispanic white *κ* =0.53; *p* = 0.446; non-Hispanic black *κ* =0.55; *p <*0.001; Non-Hispanic Asian *κ* =0.36; *p <*0.001; Hispanic *κ* =0.57; *p* = 0.209).

**Table 1:** Prevalence of metabolic syndrome and eligibility for obesity pharmacotherapy.

Characteristic	n	BMI, mean (SD), kg/m^2^	No. (%)			McNemar’s test, *p*	Cohen’s test, *κ*
			Metabolic Syndrome	FDA Eligible	Metabolic Syndrome, non-FDA Eligible		
**Total**	6,646	29.6 (7.4)	2,444 (37)	2,692 (41)	869 (36)	<0.001	0.37
Age category, y							
**18-39**	2,254	28.9 (8.1)	485 (7)	830 (12)	115 (24)	<0.001	0.40
**40-59**	2,122	34.1 (7.4)	869 (13)	950 (14)	274 (32)	0.001	0.40
**≥ 60**	2,270	35.6 (6.5)	912 (16)	912 (14)	480 (44)	<0.001	0.31
Sex							
**Men**	3,219	28.9 (6.4)	1,140 (35)	1,161 (36)	423 (37)	0.476	0.42
**Women**	3,427	30.3 (8.1)	1,304 (38)	1,531 (45)	446 (34)	<0.001	0.33
Race and Ethnicity							
**Non-Hispanic White**	2,236	29.5 (7.2)	456 (20)	442 (20)	176 (39)	0.446	0.53
**Non-Hispanic Black**	1,566	31.1 (8.6)	195 (12)	266 (17)	53 (27)	<0.001	0.55
**Non-Hispanic Asian**	810	25.3 (4.6)	97 (12)	45 (6)	68 (70)	<0.001	0.36
**Hispanic**	1,724	30.3 (6.7)	322 (19)	341 (20)	105 (33)	0.209	0.57
**Other**	310	30.2 (7.8)	70 (23)	67 (22)	21 (30)	0.631	0.63

## Discussion

The FDA approvals of phentermine-topiramate, bupropion-naltrexone, and liraglutide for obesity by 2015 ushered in a new age of therapeutic options. Now, the AMA is encouraging a more holistic assessment of individual obesity, recognizing the heterogeneity of risk among subgroups. A previous analysis of NHANES mortality data identified normal-weight MetS participants as having the highest mortality risk (13). Recently, liraglutide combined with exercise and a low-calorie diet was highly effective in the treatment of MetS (2, 3). Yet, our analysis of NHANES data demonstrates that many people with MetS may not be eligible for obesity pharmacotherapy. Body mass index thresholds impose a barrier to treatment, where metabolic syndrome, regardless of BMI, significantly increases morbidity and mortality.

Subsets of individuals such as metabolically obese normal weight (MONW) (“skinny fat”) as well as metabolically healthy obesity (MHO) make the body size metric (i.e., BMI) a specific challenge for prescribing obesity-related drugs. Variation in the relationship between body size and metabolic disease risk across race is another disadvantage to the use of BMI metrics, affecting equity of medical care. From our analyses, groups that showed the lowest levels of agreement (i.e., people ≥60 years *κ* = 0.31 and non-Hispanic Asians *κ* = 0.36) shows areas that should be investigated further to assess individual, age, or group related differences and how they related to these classification methods. This additionally highlights potential individual differences that are lost within the classifications (i.e., non-Hispanic Asian is very broad) and the complication in using single statistical approaches for aggregate data. It should be noted that a different health risk association with BMI has been previously documented for some Asian Pacific populations compared to western cohorts, where Asians have a lower BMI health risk threshold and Pacific Islanders have a higher BMI health risk threshold (14). Further, fat redistributes from subcutaneous to visceral fat with age (15). These examples highlight the need for a different or additional metric that more closely reflects intraabdominal obesity, perhaps at least inclusion of a waist/height ratio (16).

While caveats to the application of BMI thresholds have been proposed for specific groups and populations (17, 18), other studies have highlighted important differences in relative body fat or distribution in relation to disease risk (6). For example, lower visceral adipose tissue (VAT) for non-Hispanic black populations compared to others with similar BMI and WC measures (19). Significant work has been done showing such differences across race/ethnicity groups for both WC- and BMI-based thresholds of metabolic and cardiovascular disease risk (20–22). Regardless of differences observed between race/ethnicity groups, the use of a crude surrogate metric of adiposity or central adiposity (e.g., BMI, WC) instead of direct, presumably causal, measures of total adiposity or VAT, makes strict interpretation of these criteria inadequate for access to treatment. However, a recent framework proposes use of a wider aperture for diagnosis, staging and managing obesity, and provides a more objective method of classifying obesity from a health perspective than just body size (16). Busetto et al. have suggested a framework for obesity management that goes beyond treatment of only the medical domain of metabolic disease, to include a functional/physical domain as well as a psychological domain of excess fat mass (16). This would incorporate a larger segment of the general population comprised of “apparently healthy” overweight and obese individuals.” (23).

## Conclusion

Current Food and Drug Administration (FDA) guidelines using body mass index (BMI) thresholds impose a barrier to pharmacotherapy treatment that excludes a significant portion of adults with obesity-related comorbidities such as metabolic syndrome. These metabolic syndrome positive individuals, regardless of BMI, have significantly increased risk of morbidity and mortality and should be assessed in a more holistic manner.

## Data availability statement

Data from this analysis is openly available to anyone under NHANES, found here: https://www.cdc.gov/nchs/nhanes/about_nhanes.htm.

## Ethics statement

The studies involving humans were approved by Data from cross sectional sampling of the US population via the National Health and Nutrition Examination Survey (NHANES) public use datasets was used for this study. The NHANES has been approved by the NCHS Research Ethics Review Board, therefor this analysis did not require a separate regulatory approval. The studies were conducted in accordance with the local legislation and institutional requirements. The participants provided their written informed consent to participate in this study.

## Author contributions

GC: Conceptualization, Writing – original draft, Writing – review & editing. AP: Conceptualization, Data curation, Formal analysis, Writing – original draft, Writing – review & editing. KF: Conceptualization, Writing – original draft, Writing – review & editing.
