# Supplementary figures and images for: Body mass index is a barrier to obesity treatment
Source: Front Endocrinol (Lausanne). 2024 Aug 1;15:1444568. doi: 10.3389/fendo.2024.1444568 (PMC11324493; doi:10.3389/fendo.2024.1444568)

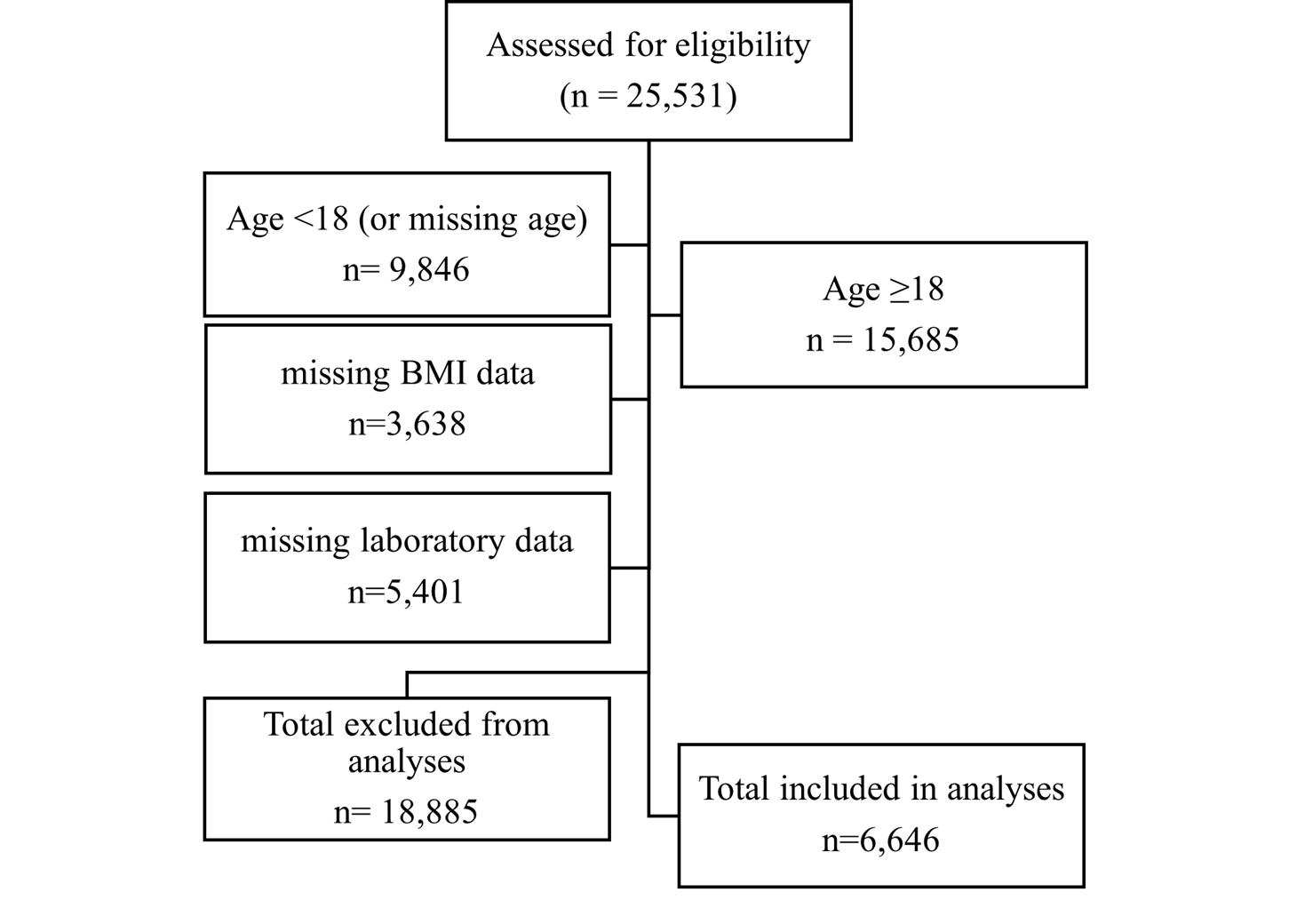

Supplement: Supplementary file 1 [file Image_1.tif]
