# Supplementary material for: Body mass index is a barrier to obesity treatment
Source: Front Endocrinol (Lausanne). 2024 Aug 1;15:1444568. doi: 10.3389/fendo.2024.1444568 (PMC11324493; doi:10.3389/fendo.2024.1444568)
Supplement: Supplementary file 2 [file Table_1.docx]

**Table S1.** Sample descriptive statistics

|  | **Men** | **Women** | **Total Sample** |
| --- | --- | --- | --- |
| **n** | 3,219 | 3,427 | 6,646 |
| **Age (y)** | 49.6 ± 18.5 | 49.0 ± 18.0 | 49.3 ± 18.2 |
| **BMI (kg/m^2^)** | 28.9 ± 6.4 | 30.3 ± 8.1 | 29.6 ± 7.4 |
| **MetSyn (n,%)** | 1,140 (35%) | 1,304 (38%) | 2,444 (37%) |
| **Non-Hispanic White** | 1,134 | 1,102 | 2,236 (34%) |
| **Non-Hispanic Black** | 719 | 847 | 1,566 (24%) |
| **Non-Hispanic Asian** | 382 | 428 | 810 (12%) |
| **Hispanic** | 823 | 901 | 1,724 (26%) |
| **Other** | 161 | 149 | 310 (5%) |
